# Penile Mondor’s Disease: Rare Association With Excessive Masturbation

**DOI:** 10.7759/cureus.8317

**Published:** 2020-05-27

**Authors:** Muhammad Mubbashir Sheikh, Hafiz Muhammad Jeelani, Aneeba Farooqi, Adeel Riaz

**Affiliations:** 1 Oncology, Northwestern University Feinberg School of Medicine, Chicago, USA; 2 Internal Medicine, Rosalind Franklin University of Medicine and Science, McHenry, USA; 3 Internal Medicine, Rosalind Franklin University of Medicine and Science, Mchenry, USA; 4 Anesthesiology and Critical Care, District Headquarter Hospital, Sahiwal, PAK

**Keywords:** penile diseases, masturbation, vigorous sexual activity

## Abstract

Penile Mondor’s disease is a rare condition characterized by thrombophlebitis of superficial penile dorsal veins. We report a case of a 26-year-old male who developed a lump on the dorsal surface of the penis following intensive masturbation after prolonged sexual abstinence. Physical examination revealed a firm, cord-like swelling with beaded texture with no localized regional lymphadenopathy. The patient was successfully managed with non-steroidal inflammatory drugs and was symptom-free on a follow-up.

## Introduction

Mondor’s disease (MD) is a self-limiting and benign condition, recognized clinically by the presence of palpable cord-like induration on the body surface. The first case was reported in 1939 as a result of generalized thoracoabdominal phlebitis and as an isolated superficial penile veins thrombosis known as Penile Mondor’s disease (PMD) in 1958 [1,2]. Other sites that can be involved are breast and axilla. Reported literature suggests that Virchow triad can explain the pathogenesis and development of PMD; vigorous sexual activity “penile trauma” leads to vessel wall damage, use of aphrodisiacs, and phosphodiesterase type 5 (PDE5) inhibitors resulting in blood stasis; and hypercoagulation resulting secondary to underlying hematological diseases or genital infections. The aim of our article is to address the early diagnosis of PMD utilizing history and physical examination. Early recognition can help to delineate the anxiety and sexual embarrassment, and give the advantage of choosing medical treatment and limits the need of surgical options.

## Case presentation

**Figure 1:**
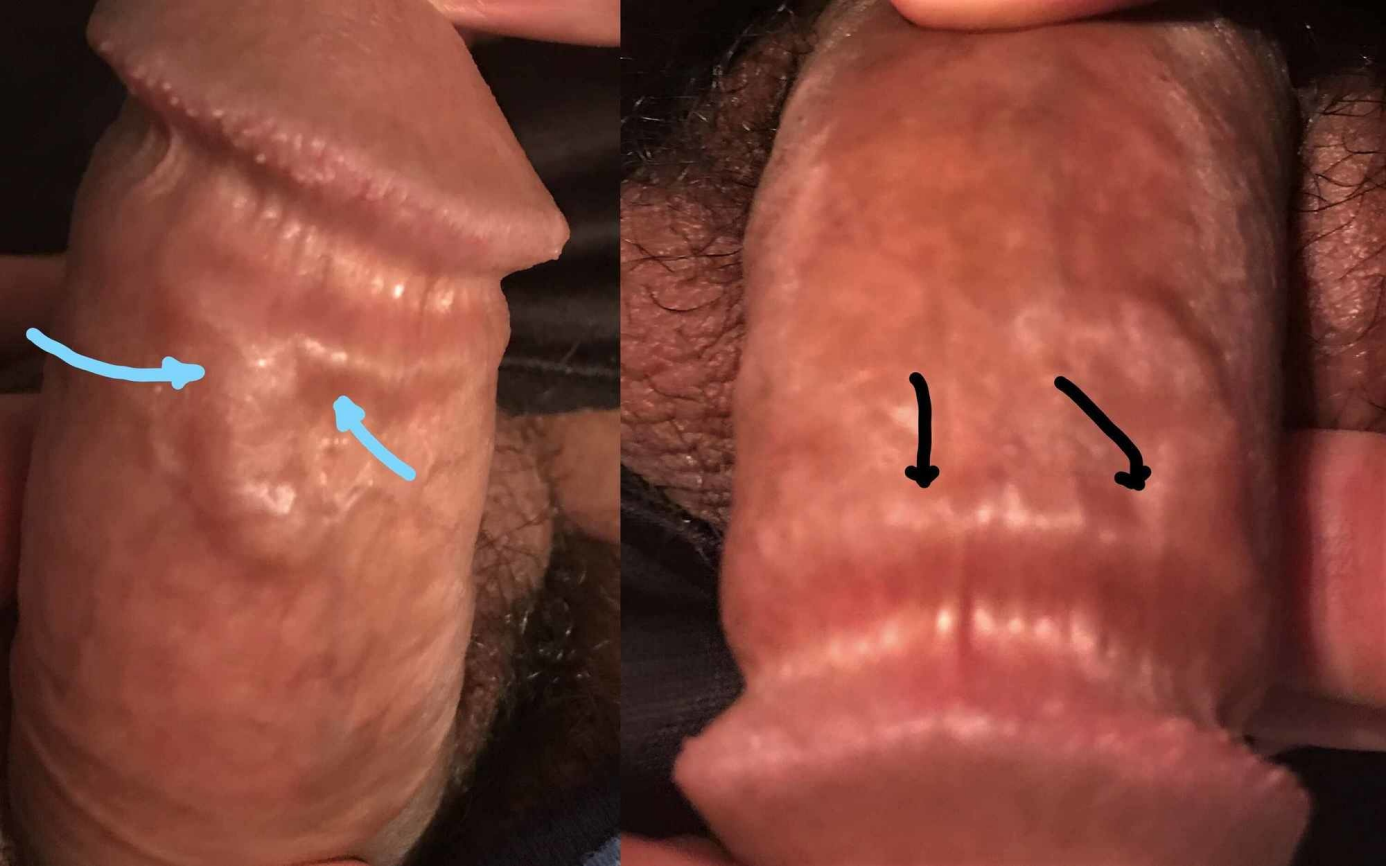
Firm cord-like swelling with beaded texture on the dorsum of the distal penis (blue arrows), extending and wrapping around the shaft just proximal to glans (black arrows).

A 26-year-old male with no significant past medical history and sexual abstinence of more than six months presented in the outpatient department with a cord-like swelling on the dorsal surface of the body of the penis for one day. He first noticed a small lump on the dorsal surface at the distal shaft that suddenly progressed to swelling, extending distally and wrapping like a cord around the shaft of the penis just proximal to glans. The patient had mild pain at rest and moderate discomfort on erection. He reported masturbating three times a day in the last seven days. He denied any recent systemic symptoms such as fever, urinary frequency/urgency, dysuria, hematuria, urethral discharge, local trauma, or any history of current or past sexually transmitted diseases. Physical examination revealed a firm cord-like swelling with beaded texture on the dorsum of the distal penis, extending and wrapping around the shaft just proximal to glans (Figure 1). No localized tenderness, erythema or skin erosions, and regional lymphadenopathy were appreciated. Screening tests for sexually transmitted diseases, urine examination, urethral culture, and inflammatory markers were negative. A color Doppler ultrasound was indicated, but declined by the patient. The patient was advised to avoid any sexual activity or masturbation for four weeks and prescribed oral ibuprofen 600 mg two times a day. The patient was pain-free within two days and penile swelling was resolved within seven days.

## Discussion

MD is a rare clinical entity with a reported incidence of 1.39%. It is described as sclerosis of superficial thrombophlebitis of the veins of the anterior thoracoabdominal wall. PMD or Mondor’s cord has been historically reported as affecting the superficial veins on the dorsal surface of the penis. Table 1 shows the most common causes of PMD [1].

**Table 1:** Most common etiologies of penile Mondor’s disease (PMD)

Infections	Surgical	Traumatic	Other
History of sexual transmitted diseases	Repair of inguinal hernia	Penile trauma	Thrombophilia
Syphilis	Orchiopexy	Use of vacuum device	Tendency to thrombosis
Candida infections	Varicocelectomy	Vigorous sexual activities or sexual abstinence	Use of intracavernous drugs

PMD is usually a clinical diagnosis. History and physical examination are reliable diagnostic tools that can reveal a palpable, firm cord-like lesion on the dorsal aspect of the penis. Doppler ultrasound can be utilized as next for differential diagnosis or if the physical examination is unclear. We used clinical judgment to diagnose our patient. The presence of thrombosed segment and uncompressible vein with no signal flow on ultrasound plus high resistance, low-velocity blood flow in cavernous arteries on Doppler are supporting features for disease and helps to differentiate it from sclerosing lymphangitis [3]. MRI use is controversial, except when closed differentials, such as Peyronie’s disease and sclerosing lymphangitis, have been ruled out [4]. However, published literature does not support additional imaging apart from ultrasonography [5].

The condition is usually self-limiting resolving within four to six weeks. Treatment is generally supportive and limited to the use of mostly non-steroidal inflammatory agents orally or anti-coagulants in topical forms, with a focus on relief of pain and inflammation [6-9]. Other measures such as warm compresses, sexual abstinence until the symptoms resolve, and patient reassurance are also found effective in relieving anxiety and embarrassment surrounding sexual tension. Refractory pain can be managed by injecting local anesthetics [10]. Surgical treatment has been implored in case of failure of medical treatment and includes thrombectomy and superficial penile vein resection. Prognosis is excellent, and no permanent long-term sequelae have been reported [11].

## Conclusions

PMD is a rare and underreported condition that manifests as cord-like swelling on the dorsal surface of the penis. Attention should be given to secondary underlying causes, such as thrombophilia, malignancy, or sexually transmitted diseases. Ultrasonography with Doppler is the imaging of choice for diagnosis, and invasive or expensive investigations should be avoided. Management should be focused on reassurance about the condition and pain alleviation. Surgery should be reserved for only refractory cases.
